# Recombinant design of the enzymatically active domain of phage Enc34 endolysin to improve its activity against Gram-negative bacteria

**DOI:** 10.1093/femsle/fnae103

**Published:** 2024-11-29

**Authors:** Tatjana Kazaka, Nikita Zrelovs, Inara Akopjana, Janis Bogans, Juris Jansons, Andris Dislers, Andris Kazaks

**Affiliations:** Latvian Biomedical Research and Study Centre, Ratsupites 1 k-1, Riga LV-1067, Latvia; Latvian Biomedical Research and Study Centre, Ratsupites 1 k-1, Riga LV-1067, Latvia; Latvian Biomedical Research and Study Centre, Ratsupites 1 k-1, Riga LV-1067, Latvia; Latvian Biomedical Research and Study Centre, Ratsupites 1 k-1, Riga LV-1067, Latvia; Latvian Biomedical Research and Study Centre, Ratsupites 1 k-1, Riga LV-1067, Latvia; Latvian Biomedical Research and Study Centre, Ratsupites 1 k-1, Riga LV-1067, Latvia; Latvian Biomedical Research and Study Centre, Ratsupites 1 k-1, Riga LV-1067, Latvia

**Keywords:** phage Enc34 endolysin, enzymatically active domain, antimicrobial peptides, Gram-negative bacteria, peptidoglycan, antibacterial tools

## Abstract

Endolysins are bacteriophage-encoded peptidoglycan-degrading enzymes with potential applications for treating multidrug-resistant bacterial infections. While exogenously applied endolysins are active against Gram-positive bacteria in their native form, Gram-negative bacteria are protected from such activity of most native endolysins by an outer membrane. However, it was shown that recombinant endolysins can be designed to efficiently lyse Gram-negative bacteria from without as well. During our previous efforts, we purified and structurally characterized the enzymatically active domain (EAD) of phage Enc34 endolysin. In this work, we investigated the lytic potential of products resulting from different variants of fusions involving this EAD with a panel of selected antimicrobial peptides. A set of constructs was generated and expressed in *Escherichia coli* cells. While most such recombinant proteins accumulated intracellularly, some of them could lyse cells from within and appear in the expression medium. The fusion protein variants produced were purified and tested for their bactericidal activity against Gram-negative bacteria. The best candidate caused rapid degradation of *E. coli* XL1-Blue cells during the first minutes after addition, reducing the viable cell count more than three-fold. We believe that these results might be helpful in the design of new antibacterial tools.

## Introduction

The tailed double-stranded DNA bacteriophages (class Caudoviricetes)—arguably the most abundant and genetically diverse virus group in nature (Camargo et al. 2022)—all appear to encode endolysins. These enzymes degrade the peptidoglycan (PG) layer within the bacterial cell wall at the end of the phage lytic cycle, resulting in host cell's death and the phage progeny particles releasing into the surrounding environment. While phages also encode other proteins with antibacterial properties, endolysins are probably the most attractive and popular candidates for the design of novel enzyme-based antibacterials, also known as “enzybiotics” (Nelson et al. 2001, Dams and Briers 2019, Kim et al. 2019). The potential of exogenously applied endolysins as human therapeutics, veterinary treatments, and food and environmental decontaminants has been documented well (Abdelrahman et al. 2021), and at least a single endolysin product that targets *Staphylococcus aureus* infections in patients with chronic skin disease (Staphefekt^TM^ SA.100, Micreos, The Netherlands) is already commercially available in Europe at the time of writing (Totté et al. 2017).

Since its conception, endolysin research has predominantly focused on Gram-positive bacteria, which lack the outer membrane (OM) layer and are thus considerably more susceptible to an exogenous application of phage lytic enzymes (Fischetti 2010). Only a fraction of natural endolysins tested against Gram-negative pathogens, such as *Acinetobacter baumanii* (Lai et al. 2011, Lood et al. 2015) and *Pseudomonas aeruginosa* (Walmagh et al. 2012), were shown to naturally permeate the bacterial OM by mechanisms that likely involve amphipathic or positively charged protein regions but are not entirely understood. Several strategies for OM destabilization and endolysin delivery to the PG layer have been proposed, which include preparations containing membrane-permeabilizing agents (Briers et al. 2011) or antibiotics (Thummeepak et al. 2016), a fusion of the endolysin to the phage receptor-binding protein (Zampara et al. 2020), as well as its encapsulation within liposomes (Bai et al. 2019). Endolysin fusions with OM-permeabilizing antimicrobial peptides (AMPs) provide another auspicious approach for endolysin-based drug design, demonstrated by the efficacy of the recently developed Artilysin® Art-175 (Lysando, Germany) against *A. baumanii, P. aeruginosa*, and colistin-resistant *Escherichia coli* (Schirmeier et al. 2018).

AMPs constitute a diverse repertoire of small molecules, most of which are between 10 and 50 amino acids in length. Many AMPs are naturally produced by different organisms and serve to either directly kill or inhibit the growth of competing microorganisms, or to modulate the innate immune response in higher organisms (Pushpanathan et al. 2013). AMPs are being actively researched regarding their potential to be used against various bacterial pathogens (Huan et al. 2020). The primary mechanism by which AMPs exert their effects involves interactions with bacterial membranes or cell walls through electrostatic interactions, followed by membrane disruption and inhibition of intracellular functions (Luo and Song 2021). As of January 2024, 3940 different AMPs were listed in the Antimicrobial Peptide Database, which likely serves as the most extensive cataloging effort for different AMPs [https://aps.unmc.edu/home, accessed on 23rd of May 2024 (Wang et al. 2016)].

The endolysins themselves are also highly diverse, and the increasing amount of next-generation sequencing data, in combination with developments in bioinformatic software tools, have uncovered a vast diversity of endolysin sequences and architectures within phage genomes. While phage endolysins are undoubtedly of varied lytic strength, host range, and resistance potential, only a tiny fraction of the alleged endolysins identified in phage sequence data bioinformatically have been experimentally characterized regarding their effects on bacterial cells (Fernández-Ruiz et al. 2018). The highly diverse phage endolysins can be combined with both natural and artificial AMPs in many different combinations, some of which are expected to be better than others at improving the bactericidal action of the endolysin in its native form. The effects of different endolysin and AMP combination variants are, however, not easily predicted in advance and require substantial experimental work to arrive at the protein with improved bactericidal properties, although high-throughput frameworks aiding the design of engineered lysins have already been proposed (Gerstmans et al. 2020).

Here, we describe the results of a step-by-step design of an unusual endolysin (Cernooka et al. 2022) partaken in an attempt to improve its exogenous activity against Gram-negative cells from without. The lytic potential of the enzymatically active domain (EAD) from phage Enc34 endolysin (protein accession number: YP_007007038.1) is evaluated both in a standalone fashion and in the form of fusion variants with a set of selected AMPs.

## Materials and methods

### Plasmid constructions

Plasmids for expression of phage Enc34 endolysin EAD (1–169 aa of YP_007007038.1), variant encoding EAD with the first transmembrane (TM) helix (1–211 aa of YP_007007038.1) as well as the full-length endolysin (241 aa, YP_007007038.1) were constructed by polymerase chain reaction (PCR)-amplification of respective fragments from phage genomic DNA and subsequent cloning in pET24a(+) vector (Novagen) using *Nde*I and *Xho*I restriction sites. All these constructs contained MGSH_6_GS sequence at the N-terminus. This artificial sequence contains six histidines for Ni-affinity purification separated by short glycine-serine (GS) linkers at both sites. The second GS-encoding sequence also contains the *Bam*HI restriction site. AMP–EAD and EAD–AMP fusions were generated by gene synthesis at BioCat GmbH (Heidelberg, Germany). Genes were subsequently incorporated into the pET24a(+) vector using *Nde*I and *Xho*I restriction sites.

### Expression conditions

Expression plasmids were transformed in *E. coli* strain BL21 (DE3) cells. For inoculum, the cells were cultivated without shaking overnight at +37°C in a selective lysogeny broth (LB) medium (10 g/l tryptone, 5 g/l yeast extract, and 10 g/l NaCl, pH 7.2) containing 30 µg/ml kanamycin. To induce protein synthesis, the cells were transferred to 400 ml of selective 2xTY medium (16 g/l tryptone, 10 g/l yeast extract, and 5 g/l NaCl, pH 7.2) with the following additives: 60 ml of phosphate solution (23.13 g KH_2_PO_4_, 125.42 g K_2_HPO_4_, and dH_2_O until 1 l, pH 7.4), 0.75 ml of 40% glucose, 1 ml of 80% glycerol, 5 ml of 10% lactose, and 0.4 ml of 1 M MgSO_4_. Expression was done in Erlenmeyer flasks on a shaker with 200 rpm at +37°C overnight. After low-speed centrifugation, cell and medium fractions were collected and analyzed for the presence of target protein.

### Protein purification

For intracellular protein purification, 1 g of wet cells was disrupted in 6 ml of lysis buffer (20 mM Tris–HCl and 300 mM NaCl, pH 8.0) by sonication, and soluble proteins were separated after centrifugation for 30 min at 18 500 x *g*. The proteins were purified using metal–ion affinity chromatography on the HiTrap IMAC FF column (Cytiva) with HIS-Select Nickel Affinity Gel. The supernatant was applied (1 ml/min) on a column containing 1 ml gel equilibrated with a lysis buffer. After washing the column with 10–20 ml of lysis buffer with 10 mM imidazole, the protein was eluted with elution buffer (20 mM Tris–HCl, 0.5 M NaCl, and 0.5 M imidazole, pH 8.0) in 1 ml fractions using linear gradient (10 CV).

Alternatively, for purification of extracellular protein, 400 ml of expression medium without cells was passed through HisTrap excel 5 ml column (Cytiva) in lysis buffer at 5 ml/min and washed with 50 ml of 10 mM imidazole buffer. The target protein was collected as one 10 ml fraction with elution buffer. Purification was performed using the Akta Prime Plus system (Cytiva).

Purified proteins were then transferred to phosphate buffered saline (PBS) and concentrated to 2 mg/ml stock solutions using an Amicon 10 kDa MWCO filter device (Millipore) and stored at +4°C until use.

### Activity assay

To evaluate the effects of our recombinantly produced EAD-containing product variants on Gram-negative bacteria, *E. coli* strain XL1-Blue was chosen to serve as a model organism. For a rough first activity check, endolysin solution at 10 µg/ml was added to growing cells at optical density (OD) ∼0.2 (A_540_), cultivated in tubes with shaking at +37°C. OD was measured 2–5 h post endolysin challenge and compared with control growth curve.

For a more precise test, bacterial cells (*E. coli, Pantoea agglomerans*, and *Hafnia alvei*) were cultivated without shaking in LB medium at +37°C overnight and collected by low-speed centrifugation. Cells were washed with PBS once, centrifuged, and resuspended in PBS until OD of 1.0 (A_540_). The cell suspension was next aliquoted in multiple tubes by 1 ml, and 5 µl of the selected recombinantly produced product solution was added until the final concentration reached 10 µg/ml (corresponding to 0.41–0.43 µM concentrations for constructs tested). EAD alone and lysozyme were used as controls. In the control tubes, the same volume of PBS was added. Prior to the start of the experiment and after defined time points (5, 20, 40, and 60 min after mixing the cells with a product), the aliquots of the cell suspension were taken and immediately used for the preparation of the serial dilutions up to 10^5^ with a step of 10^1^ using PBS, 50 µl of which were spread on LB agar plates afterward. The viable cell titer (CFU/ml) in the samples at the given time points after the addition of the product was derived from the number of colony-forming units observed after overnight incubation at +37°C, the dilution factor, and the volume of the dilution used. Product activity assays were performed in triplicate.

The viable cell count titer reduction (% of CFU/ml) alleged to the bactericidal activity of a product was evaluated at each different time point post-addition as follows: average viable cell titer in the respective treatment group was divided by an average cell titer in the control group and the percentage of change was obtained by subtracting this fraction from 100% (representing control group).

### Electron microscopy

*Escherichia coli* cells were absorbed from the suspension onto carbonized formvar-coated 300 Mesh copper grids (Agar Scientific) through a 5-min incubation. Subsequently, the grids underwent rinsing with a 1 mM ethylenediaminetetraacetic acid (EDTA) solution at pH 8.0, staining with a 0.5% uranyl acetate solution for 1 min, and air drying. The stained grids were analyzed using a JEM-1230 electron microscope (JEOL) operating at an accelerating voltage of 100 kV, and images were captured with the MORADA digital camera using iTEM imaging software (Olympus).

## Results and discussion

### Lytic properties of Enc34 endolysin variants

**Figure 1.**
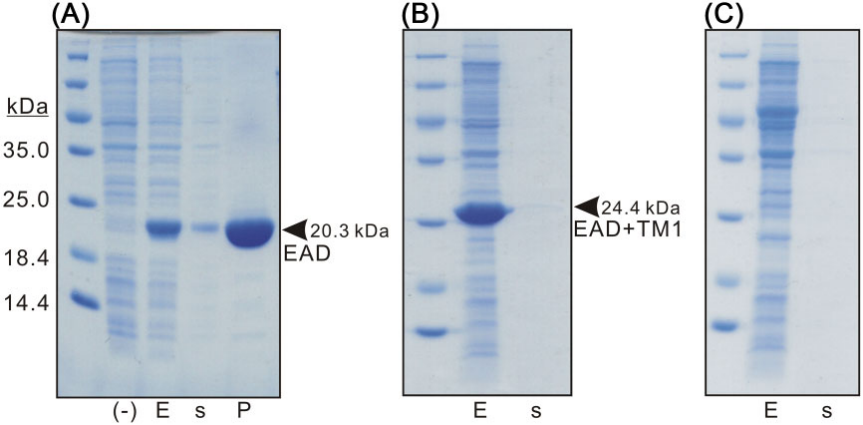
Coomassie-stained sodium dodecyl sulfate (SDS) polyacrylamide gel electrophoresis (PAGE) with 5% stacking and 15% separating gel illustrating synthesis and expression of phage Enc34 endolysin and its derivates. Tile (A)—EAD alone; tile (B)—EAD extended until aa 211; and tile (C)—full-length protein. Within the tiles, the leftmost track represents a marker, (-)—BL21 (DE3) cells that served as a negative control; E—target protein expression in cells (whole cell lysate); s—expression medium after sedimentation of cells; and P—protein purified from the expression medium.

Phage Enc34 was the first phage that has been isolated and completely sequenced at our lab (Kazaks et al. 2012, Cernooka et al. 2024), and ORF39 in its genome encodes a novel type of endolysin with an uncharacterized EAD and two C-terminal TM regions. In our previous study, the EAD of phage Enc34 endolysin was expressed, purified, and its structure was solved (Cernooka et al. 2022). Also, EAD PG-degrading activity on Gram-negative OM-permeabilized cells has been demonstrated, while the lytic effect on intact cells was not observed (Cernooka et al. 2022). Here, in the frames of an ongoing project, we aimed to investigate in detail the lytic potential of Enc34 endolysin variants alone or in combination with a set of AMPs on intact Gram-negative bacteria. We continued our experiments with EAD expression in *E. coli* and surprisingly found that EAD alone, without TM domains, is capable of cell lysis from within, which is confirmed by the presence of protein in the expression medium. This protein can be easily purified until near homogeneity by single-step Ni-affinity chromatography (Fig. 1A). We then reasonably assumed that the presence of TM domain(s) in connection with EAD might enhance the lysis effect; however, an extension of EAD with the first TM domain including loop region (until aa 211), in contrast, decreased lysis from within as less protein was found in expression medium (Fig. 1B). As observed previously and in this study, expression of full-length endolysin firmly abolished cell growth and protein was not detectable either in cells or in the expression medium [(Cernooka et al. 2022); Fig. 1C]. All optimization experiments, including growth and induction time, cultivation temperature, and change of expression host to yeast, were unsuccessful in obtaining full-length protein (data not shown). We conclude that the second TM domain is toxic to cells, apparently through interaction with membranes, and even a small amount of full-length protein is enough to inhibit further synthesis. These data led us to choose a single EAD domain for further experiments involving fusions with AMPs and their characterization.

### Design and properties of EAD–AMP fusions

**Figure 2.**
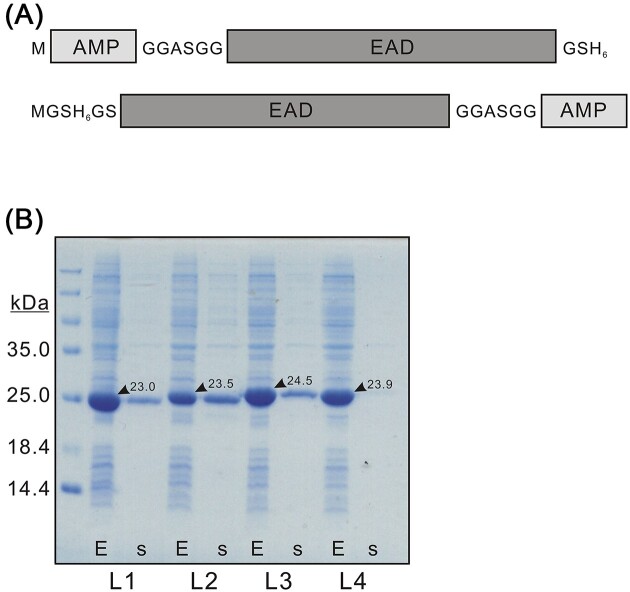
Tile (A)—design of EAD–AMP fusion constructs. Each AMP was fused to EAD either N-terminally (top) or C-terminally (bottom). Short GGASGG linker (default linker, denoted as linker 1 or L1 throughout the text) was used between the fusion parts. Tile (B)—Coomassie-stained PAGE indicating the influence of a linker sequence on the AMP7–EAD construct activity on *E. coli* cells from within. E—whole cell lysate and s—expression medium. L1–L4 denotes different linker sequences used in constructs.

In the first part of the experiments, EAD was fused with a peptide from the set of considered AMPs (10 different AMPs in total; see Table 1 for details) at either the N- or C-terminus, separated by flexible linkers (Fig. 2A). All the constructs were expressed in *E. coli* BL21 (DE3) cells and purified either from cells or from the expression medium using single-step Ni-affinity chromatography as described in the “Methods” section. It should be noted that all the constructs were not generated simultaneously but in logical step-by-step portions.

**Table 1.** A list of natural and artificial AMPs used in this study.

Abbreviation (Origin)	Sequence	Reference
AMP1 (artificial)	RRRWRKRRWWW	(Yang et al. 2018)
AMP2 (artificial)	PMARNKKLLKKLRLKIAFK	(Fensterseifer et al. 2019)
AMP3 (artificial)	GFCWYVCARRNGARVCYRRCN	(Elliott et al. 2020)
AMP4 (artificial)	GIGKFLKKAKKFGKAFVKILKK	(Maloy and Kari 1995)
AMP5 (natural)	GLRKRLRKFRNKIKEKLKKIGQKIQGLLPKLAPRTDY	(Travis et al. 2000)
AMP6 (natural)	GLKEIFKAGLGSLVKGIAAHVAS	(Conlon et al. 2010)
AMP7 (artificial)	GSKKPVPIIYCNRRSGKCQRM	(Zhang et al. 2020)
AMP8 (natural)	GKGIKFVGEEIRRKSGKSAGAK	(Ma et al. 2022)
AMP9 (natural)	SFPFFPPGICKRLKRC	(Wu et al. 2011)
AMP10 (natural)	KGIVAERILKPCVRRKVNGKFRS	(Houston et al. 2022)

Artificial origin means that the sequence was not a naturally occurring one, but rather a result of design.

Among the large number of AMPs described in literature, we have selected a set of those matching most of the following criteria: (i) being maximally short (from 11 to 23 aa, with the sole exception of 37 aa-long AMP5), (ii) carrying strong cationic or amphipathic sequences, (iii) being noncytotoxic to mammalian cells, and (iv) exhibiting antimicrobial activity preferably against Gram-negative bacteria. For example, the cationic antimicrobial avian β-defensin CAMp-t2, which was modified to a short α-helical peptide RRRWRKRRWWW (AMP1), has demonstrated a solid broad-range antibacterial activity at physiological concentrations and was minimally cytotoxic to mammalian cells (Yang et al. 2018). Alternatively, an artificial cationic peptide PMARNKKLLKKLRLKIAFK (AMP2) has been reported to possess a robust antimicrobial activity selectively against Gram-negative bacteria (Fensterseifer et al. 2019). EAD fusions with AMP1 and AMP2 were generated and analyzed in the first part of the experiments. We observed that, depending on fusion type, constructs were highly different in terms of synthesis, solubility, degradation, outcome, and activity (Table 2). The activity of the proteins was assessed either from within (by the appearance of a given protein in the expression medium) or from without (by influence on growing *E. coli* cells in culture). Among these four constructs tested, no suitable candidate for efficient targeting of Gram-negative bacteria was found. The next four constructs using AMP3 and AMP4 were also not promising (Table 2) either due to strong degradation (AMP3) or due to a low expression level and solubility (AMP4). Experiments then were extended to six EAD fusion constructs with AMP5, AMP6, and AMP7. Here, the situation appeared quite different as constructs with well-detectable activity from within were noticed for the first time. The first such construct, despite low synthesis and solubility, was EAD–AMP6 (interestingly, the same fusion with AMP6 at the N-terminus showed low activity). Two other constructs were both EAD fusions with AMP7 (with AMP at either N- or C-terminus of a protein, respectively). Here, all the tested parameters, including inhibition of cell growth, matched the proposed eventual tools against Gram-negative bacteria.

**Table 2.** Properties of phage Enc34 endolysin EAD–AMP fusion constructs.

Construct	Expression^a^	Solubility^b^	Degradation^c^	Activity from within^d^	Activity from without^e^
AMP1–EAD	High	High	No	No	Negligible
EAD–AMP1	High	Low	No	No	ND
AMP2–EAD	High	High	High	No	Negligible
EAD–AMP2	High	High	Moderate	Low	Negligible
AMP3–EAD	High	High	Moderate	Moderate	Negligible
EAD–AMP3	High	Moderate	High	No	Negligible
AMP4–EAD	Low	Low	No	No	ND
EAD–AMP4	Negligible	No	ND	No	ND
AMP5–EAD	High	Low	ND	No	ND
EAD–AMP5	High	Moderate	High	Low	Negligible
AMP6–EAD	Moderate	Low	ND	Low	ND
EAD–AMP6	Low	Low	No	High	Moderate
AMP7–EAD	High	High	No	High	Moderate
EAD–AMP7	High	High	No	High	Moderate
AMP8–EAD	High	High	No	High	Moderate
EAD–AMP8	High	High	No	High	Moderate
AMP9–EAD	High	High	Moderate	High	Moderate
EAD–AMP9	High	High	Moderate	High	Moderate
AMP10–EAD	High	High	Moderate	High	Moderate
EAD–AMP10	High	High	No	High	Moderate

^a–d^Assessed by sample analysis in Coomassie-stained PAAG.

^e^Tested by *E. coli* growth inhibition in test tubes with shaking. ND, not determined.

Generally, lysis activity from within appeared very similar for the three constructs selected and correlated well with activity from without using purified products from the expression medium. However, regarding the expression level, both AMP7–EAD constructs were superior to EAD–AMP6. AMP7, or an AMP TS, is synthesized by substituting an amino acid T to S for thanatin GSKKPVPIIYCNRRTGKCQRM, which is isolated from the hemipteran insect *Podisus maculiventris* (Fehlbaum et al. 1996). It was reported that TS without hemolytic activity disrupts the integrity of the outer bacterial cell membrane and can permeabilize also the inner membrane of *E. coli*. AMP TS binds with DNA in a concentration-dependent manner (Zhang et al. 2020).

### Attempts to improve AMP7–EAD constructs

Since both AMP7–EAD and EAD–AMP7 fusions were found promising for cell growth inhibition, we wanted to test some AMP7-related peptides for similar purposes. A thorough search in the AMP database (Wang et al. 2016) revealed a peptide sequence (AMP8) with the highest percentage of similarity (38.5%) to AMP7 and matching most of the criteria described in Chapter “Design and properties of EAD–AMP fusions.” In addition, two other peptides with similarity percentages of 34.8% (AMP9) and 34.6% (AMP10) were selected. This led to the generation of another set of six constructs, which were processed in the same manner as previously. While all of the constructs from this set exhibited high synthesis and solubility, some unfortunately showed moderate levels of degradation (AMP9–EAD, EAD–AMP9, and AMP10–EAD, see Table 2). The other three were promising in terms of activity either from within or from without; however, none of them inhibited *E. coli* cell growth to such a high extent as AMP7–EAD (not shown). Thus, the AMP7–EAD construct was selected for further manipulation.

Next, we hypothesized that the linker sequence separating AMP and EAD might play a role in lytic activity, probably by making both domains more easily accessible. Therefore, for this particular construct, several flexible linker sequences were compared. These included GGASGG, linker 1/L1 (default; this study); (G_4_S)_3_, linker 2/L2 (Chen et al. 2013); KESGSVSSEQLAQFRSLD, linker 3/L3 (Bird et al. 1988); and EGKSSGSGSESKST, linker 4/L4 (Bird et al. 1988). The cells were cultivated in expression conditions, and the best activity from within was observed for construct containing linker 2, while for linker 4 it was negligible, demonstrating that linker sequence *per se* might have a dramatic effect on overall fusion protein properties (Fig. 2B).

**Figure 3.**
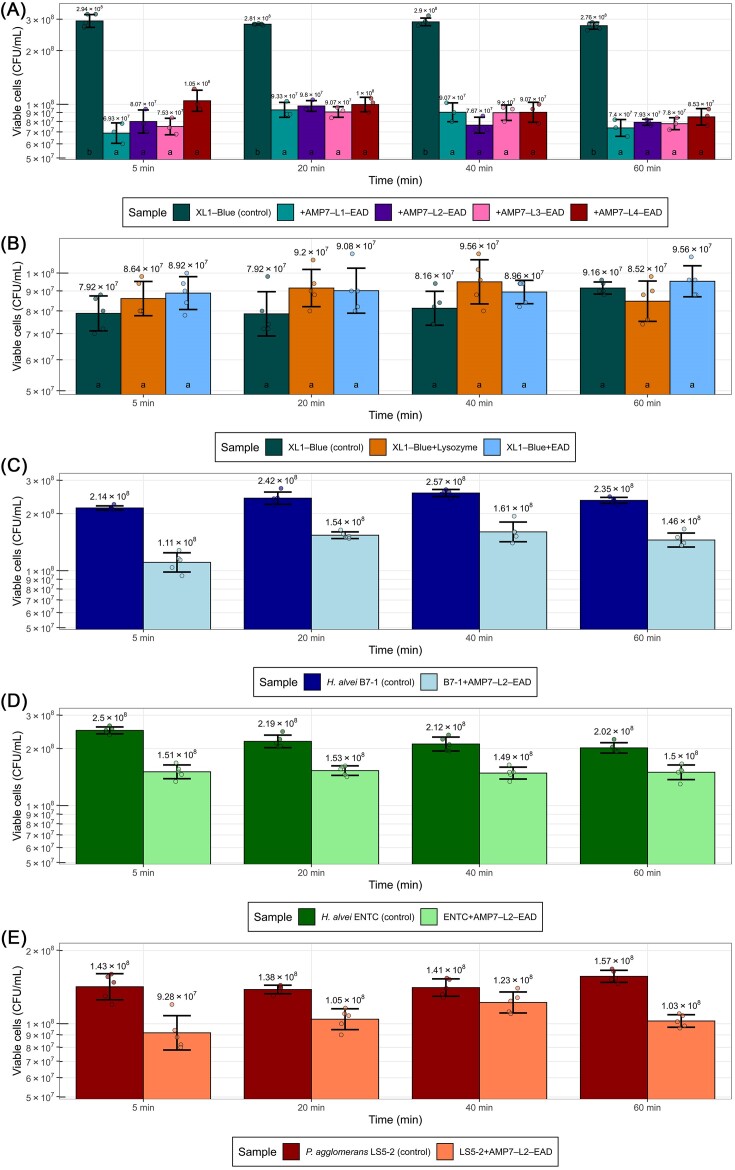
Impact of the Enc34 EAD variants on the viable bacterial cell titer. Tile (A)**—**lack of significant differences in bactericidal activity of AMP7 and phage Enc34 fusion constructs with different linkers tested between. Tile (B)**—**lack of significant effect from the exogenous application of either phage Enc34 EAD or lysozyme to *E. coli* XL1-Blue cells. Tile (C)**—**effects of the AMP7-L2-EAD construct addition on the *H. alvei* strain B7-1. Tile (D)**—**effects of the AMP7-L2-EAD construct addition on the *H. alvei* strain ENTC. Tile (E)**—**effects of the AMP7-L2-EAD construct addition on the *P. agglomerans* strain LS5-2. In all of the tiles, the numbers above the bars indicate an average CFU/ml for the respective group. Bar heights correspond to the mean of at least three replicates, and error bars indicate ± one standard deviation. Jittered points represent values from individual measurements of the respective group. Letters at the base of the bars in tiles (A and B) indicate grouping based on the results of Tukey's honest significance test (tested between all the experimental groups at 5, 20, 40, and 60 min separately) at alpha = 0.05. All the differences between control and treatment groups in tiles (C, D, and E) were statistically significant at alpha = 0.05 (ANOVA *P* < .05, tested at 5, 20, 40, and 60 min separately). In all the tiles, the *y*-axis is log-scaled. Note the differences between the *y*-axis limits between the tiles.

All four fusion proteins, including linkers L1–L4, were then purified from the expression medium as described in the “Methods” section and attempted to lysis from without. Equal portions of protein (5 µl from stock solution 2 mg/ml) were added to *E. coli* cells until the desired concentration was 10 µg/ml and the number of viable cells was calculated after defined time intervals by direct plating (Fig. 3A). Notably, the assay was performed without EDTA. As a result, the majority of the lytic effect was observed within the first 5 min of incubation and a further decrease in viable cell count was practically undetectable. Surprisingly, despite the highly different amounts of target protein in the expression medium (representing the outcome of purified protein 20.0, 42.3, 18.1, and 3.4 mg from 400 ml of expression medium, respectively), no statistical differences in viable cell number were observed between constructs indicating that activity from within not always is correlated to activity from without. From these data, we concluded that no further improvement of a particular construct could be achieved with the utilized “building blocks” and methodology employed for this study. As expected, the exogenous application of the EAD domain alone, similar to the application of the lysozyme, did not affect the viable cell number compared to an untreated negative control (Fig. 3B).

Additionally, we have tested the activity of the AMP7-L2-EAD (N-to-C) construct against some other wild-type Gram-negative bacterial species and strains previously isolated in our lab from insects. The observed effects were lower than for *E. coli* lab strain XL1-Blue, which demonstrated an average drop in CFU/ml between 64%–76% when compared to control throughout the different time points within the assays but were quite different in terms of viable cell count reduction in comparison to the control among the strains tested (Fig. 3C–E). When AMP7-L2-EAD was applied against *H. alvei* strain B7-1 (host of phages Pocis76 and Pocitis76 available from GenBank under accessions MW689258.1 and OL512804.1, respectively), which bacteriophage Enc34 could not infect, the reduction in average viable cell counts across the different time points was between 38% and 48% (Fig. 3C).

When applied against *P. agglomerans* LS5-2 [host of bacteriophages Nifs112, Nufs112, and Nafs113 recently described (Zrelovs et al. 2023)], the average reduction in viable CFU/ml was ~35% both after 5 min, as well as after an hour post-addition (Fig. 3D).

When tested against the isolation host of bacteriophage Enc34 (from whose endolysin the EAD used in this study was derived)—*H. alvei* strain ENTC (Cernooka et al. 2024), the construct has shown a reduction in the average titer of viable cells of 26%–40%, depending on the time point since addition (Fig. 3E).

**Figure 4.**
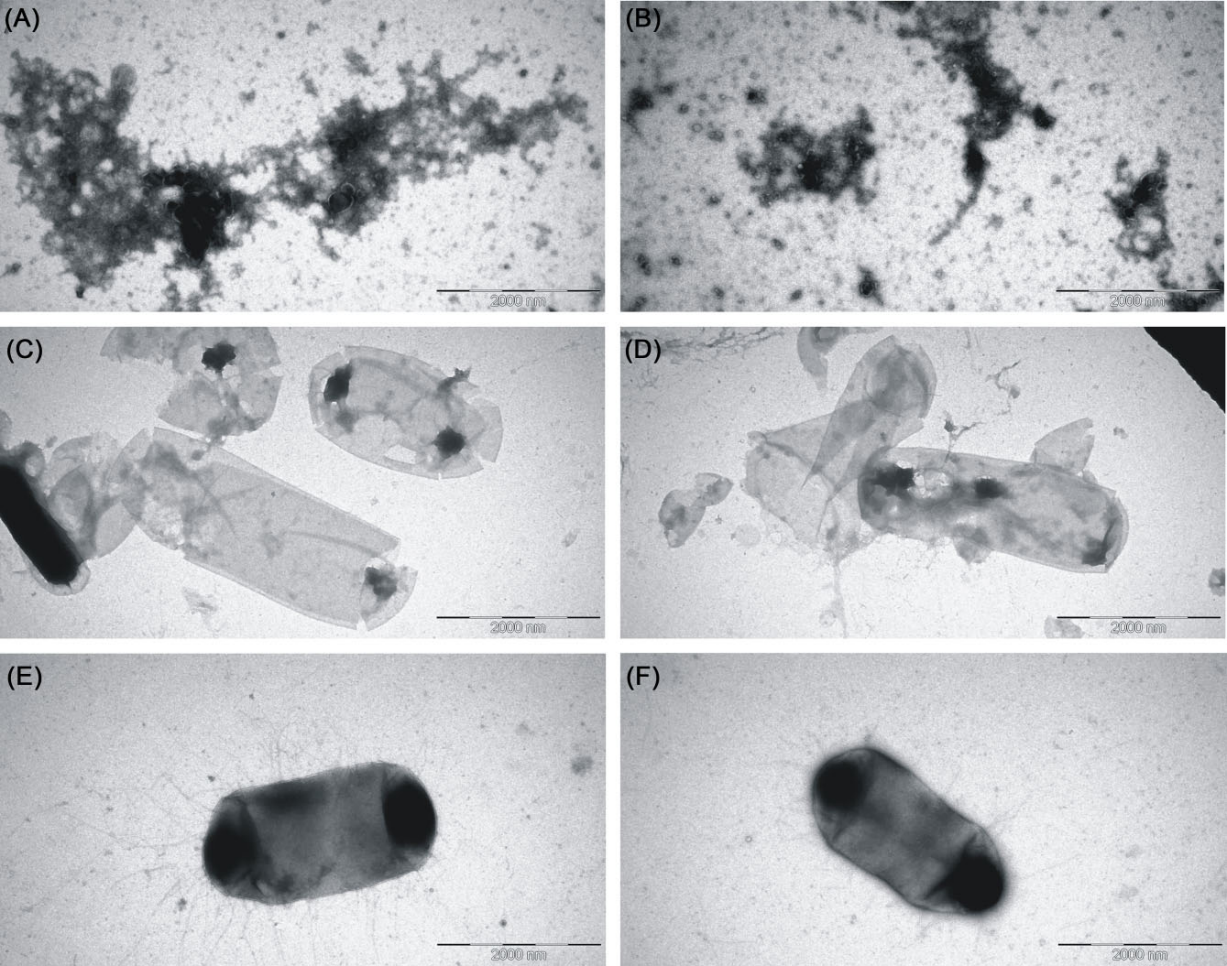
Transmission electron micrographs demonstrating the effects of the AMP7–EAD product on the integrity of *E. coli* cells. Tiles (A and B)—sonicated cells (positive control); tiles (C and D)—AMP7–EAD-treated cells; and tiles (E and F)—negative control.

At the end of incubation, *E. coli* cell samples were subjected to electron microscopy. While the majority of the cells in the endolysin-treated samples appeared damaged and demonstrated huge holes, cells in the control samples exhibited the morphology expected of the viable cells (Fig. 4). Inspection of the samples using TEM, however, hinted that remnants of the disintegrated cells seemed to adsorb onto the formvar/carbon grids better than the intact cells giving an impression that nearly all treated cells in the sample were strongly affected.

## Conclusion

A set of AMP peptide fusions with the EAD of an unusual endolysin of the phage Enc34 was tested for the construction of novel enzybiotics potentially applicable against Gram-negative bacteria. Only a limited number of constructs exhibited the desired properties regarding expression level, solubility, lack of product degradation, and acceptable activity. Among them, the AMP7–EAD construct was superior and was selected for further improvement by optimization of linker sequences between AMP and EAD parts. While these changes had a dramatic impact on activity from within, activity from without did not seem to be affected much by the use of a linker variant. The bactericidal effect of the constructs appeared within the first 5 min of co-incubation and resulted in a significant decrease in the number of viable cells; however, the total clearance of bacteria was not observed with either of the constructs. The selected AMP7-L2-EAD construct could reduce the number of viable bacterial cells of several Gram-negative bacteria from the order *Enterobacterales* it was tested against, but to a very different extent. This indicates that further improvements in the design of the most prospective candidate construct could be sought to obtain a more potent cytotoxic effect. We hope that our data will add to the global knowledge available for combating antibiotic-resistant Gram-negative bacterial pathogens and will inspire more ideas for the design of efficient alternative weapons targeted against them.
